# Red and processed meat consumption and colorectal cancer risk: a systematic review and meta-analysis

**DOI:** 10.18632/oncotarget.20667

**Published:** 2017-09-06

**Authors:** Zhanwei Zhao, Quanxin Feng, Zifang Yin, Jianbo Shuang, Bin Bai, Pengfei Yu, Min Guo, Qingchuan Zhao

**Affiliations:** ^1^ Xijing Hospital of Digestive Diseases, The Fourth Military Medical University, Xi′an, China; ^2^ Department of Obstetrics, Northwestern Women and Children’s Hospital, Xi′an, China; ^3^ Department of General Surgery, Chinese PLA 323 Hospital, Xi′an, China

**Keywords:** nutrition, red meat, processed meat, colorectal cancer, meta-analysis

## Abstract

The associations between red and processed meat consumption and the risk of colorectal cancer types have not been conclusively defined. We performed a systematic review and meta-analysis to analyze these associations. We searched PubMed and EMBASE to identify studies published from inception through September 2016. Dose-response, subgroup and subtype analyses of colorectal cancer (colon cancer, proximal colon cancer, distal colon cancer and rectal cancer) were performed. We ultimately selected 60 eligible studies. Positive associations were observed for colorectal cancer in case-control studies (red meat, *P*<0.01; processed meat, *P*<0.01) and cohort studies (red meat, *P*<0.01; processed meat, *P*<0.01). However, subtype analyses yielded null results for distal colon cancer in case-control studies (*P*=0.41) and cohort studies (*P*=0.18) for red meat and null results for proximal colon cancer in case-control studies (*P*=0.13) and cohort studies (*P*=0.39) for processed meat. Additionally, although the results of case-control studies were positive (red meat, *P*<0.01; processed meat, *P*=0.04) for rectal cancer, there were no positive associations between red (*P*=0.34) and processed meat (*P*=0.06) consumption and the risk in cohort studies. In a systematic review and meta-analysis, we found consumption of red and processed meat was associated with the risk of overall colorectal cancer but not rectal cancer. Additionally, there were no associations between the consumption of red meat and distal colon cancer risk and between the consumption of processed meat and proximal colon cancer risk.

## INTRODUCTION

According to GLOBOCAN 2012, colorectal cancer (CRC) is the second most frequently diagnosed cancer in females and the third most frequent in males, with an estimated 693,900 deaths worldwide each year [1]. Considering the increasing trend in the incidence and the high fatality, there is an urgent need to find novel strategies to prevent CRC. An increasing number of epidemiologic and clinical studies have focused on dietary factors [2, 3]. When cooked at high temperature for a long time, red and processed meats are a major source of carcinogens, including polycyclic aromatic hydrocarbons, heterocyclic amines and N-nitroso compounds, which may play a role in the development of CRC [4, 5]. Although the continuously updated report from the World Cancer Research Fund (WCRF, which is based on prospective studies published through 2010) on CRC judged the evidence for the role of red meat and processed meat to be “convincing” (http://wcrf.org/int/research-we-fund/continuous-update-project-findings-reports/colorectal-bowel-cancer), there was insufficient independent evidence on proximal colon cancer (PCC), distal colon cancer (DCC) and rectal cancer (RC). Many high quality studies have been published in recent years and thus an updated meta-analysis of the literature will likely clarify the impact of these recent studies.

Considering the large burden of CRC worldwide and the lack of sufficient evidence for the role of red and processed meats in CRC incidence, we conducted a systematic review and an updated meta-analysis with the following objectives: (1) to provide an update based on increased available evidence and a quantitative analysis of the eligible data on the associations between red and processed meat consumption and the risk of CRC, PCC, DCC and RC; (2) to provide more detailed evidence through subgroup analyses of cohort studies including geographic area, sample size, publication year, quality score and adjustments; and (3) to evaluate the dose-response association between red and processed meat consumption and CRC risk.

## RESULTS

### Literature selection, study characteristics and quality scores

**Figure 1:**
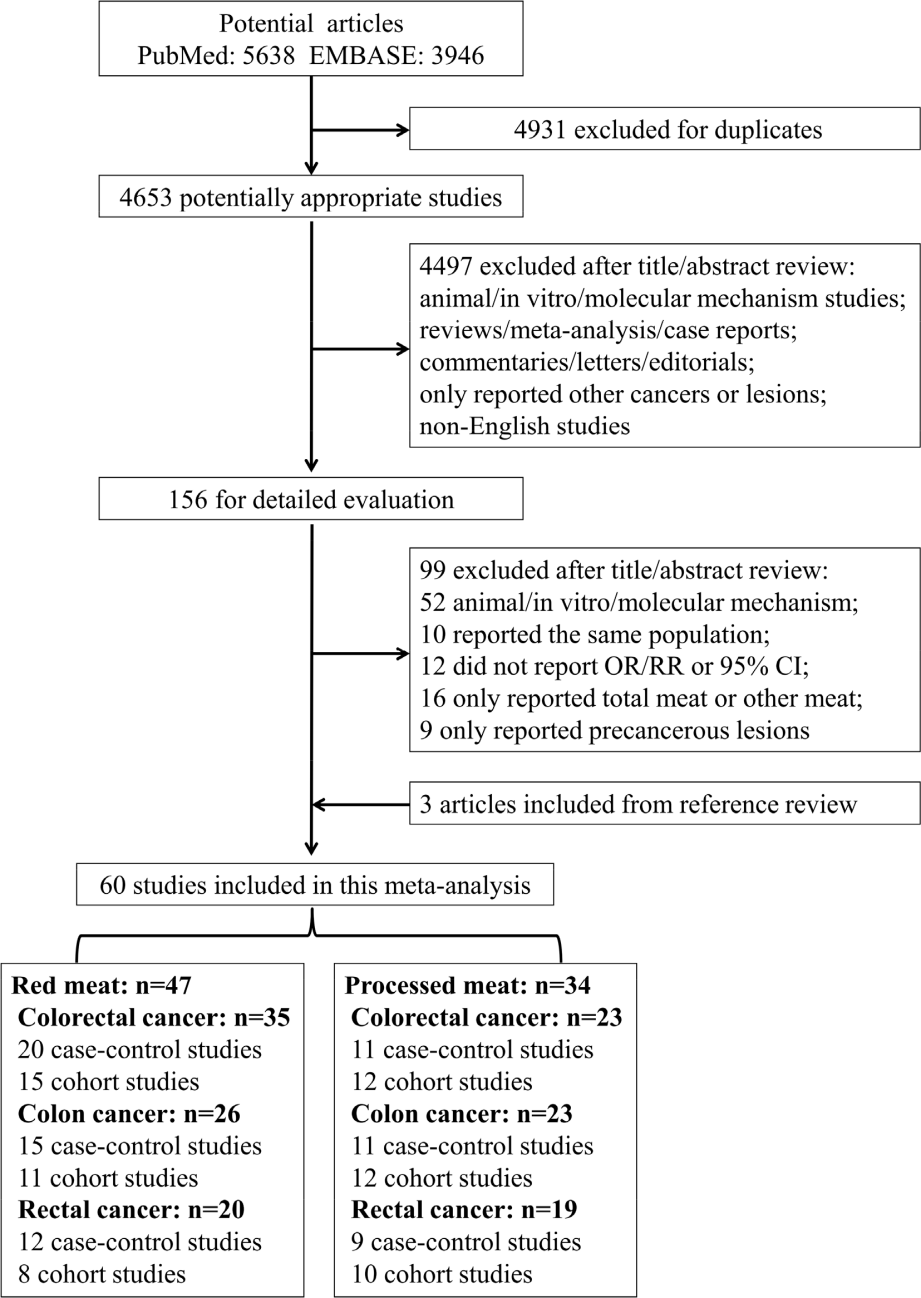
Flowchart of the process for the identification of relevant studies

Sixty studies met the eligibility criteria (Figure 1) and provided 81 separate estimates (red meat, 47; processed meat, 34) of the associations between red and processed meat consumption and the risk of CRC types. The selected studies were from 20 countries or regions in America, Europe, Asia and Australia with 1,649,315 participants and 36,843 cases for red meat consumption and 1,892,692 participants and 35,165 cases for processed meat consumption regarding CRC.

### Red meat-CRC

#### High vs low consumption

**Figure 2:**
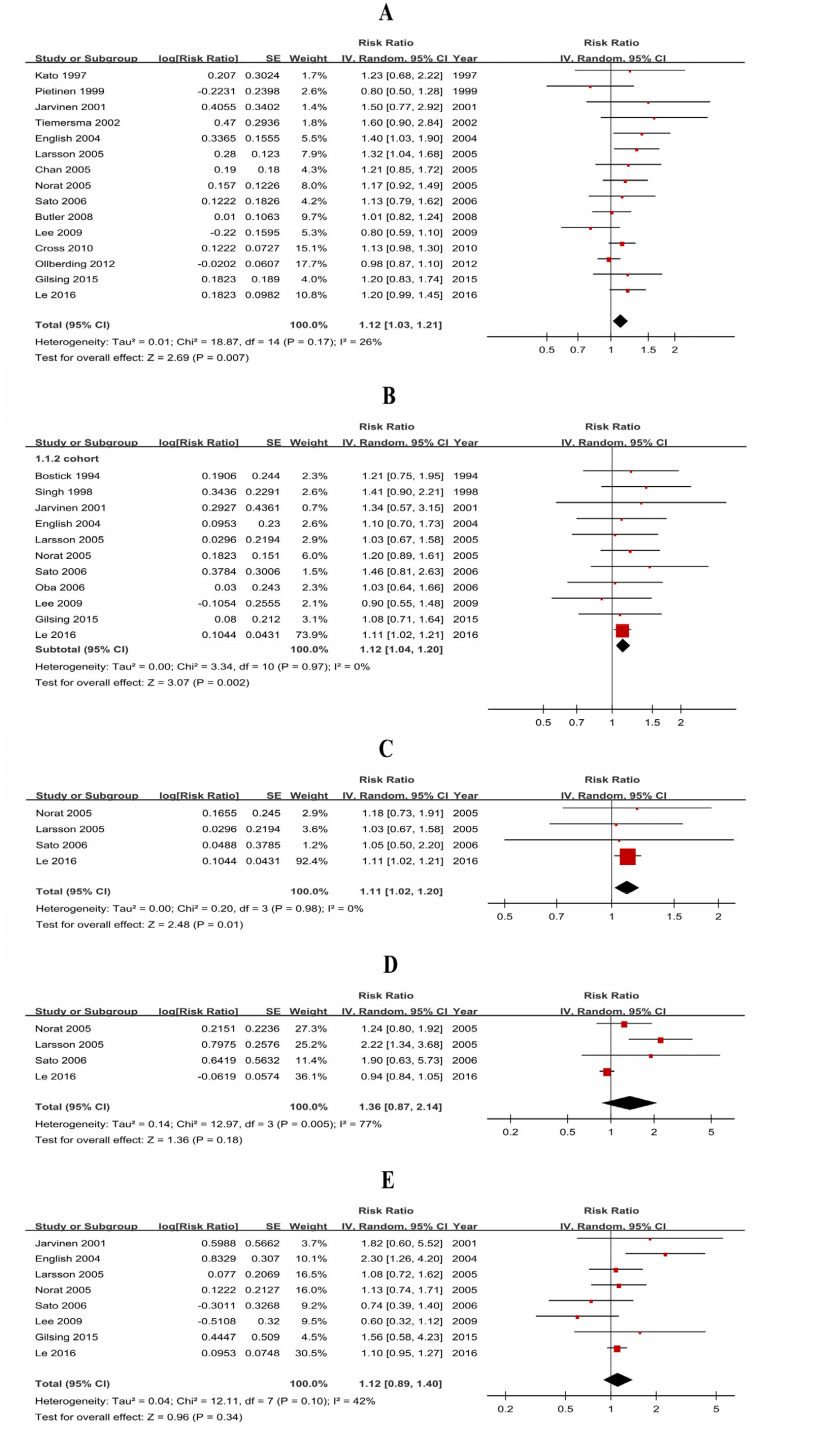
Forest plots of cohort studies for red meat consumption (highest vs lowest) and colorectal cancer risk **(A)** Colorectal cancer; **(B)** colon cancer; **(C)** proximal colon cancer; **(D)** distal colon cancer; **(E)** rectal cancer.

Thirty-five studies (20 case-control studies and 15 cohort studies) were included and the pooled RRs were 1.41 (1.17-1.71) for case-control studies (Supplementary Figure 1A) and 1.12 (1.03-1.21) for cohort studies (Figure 2A, Table 1).

**Table 1:** Subtype analyses of cohort studies for red and processed meat consumption and colorectal cancer risk

Subtypes	Red meat					Processed meat				
	n	RR (95% CI)	*P*	*P*_h_	*I*^*2*^ (%)	n	RR (95% CI)	*P*	*P*_h_	*I*^*2*^ (%)
**CRC**	15	**1.12 (1.03-1.21)**	**<.01**	.17	26	12	**1.15 (1.07-1.24)**	**<.01**	.18	27
**CC**	11	**1.12 (1.04-1.20)**	**<.01**	.97	0	12	**1.21 (1.13-1.31)**	**<.01**	.47	0
**PCC**	4	**1.11 (1.02-1.20)**	**.01**	.98	0	6	1.06 (0.92-1.23)	.39	.67	0
**DCC**	4	1.36 (0.87-2.14)	.18	<.01	77	6	**1.34 (1.15-1.56)**	**<.01**	.59	0
**RC**	8	1.12 (0.89-1.40)	.34	.10	42	10	1.17 (0.99-1.38)	.06	.07	44

CRC: colorectal cancer; CC: colon cancer; PCC: proximal colon cancer; DCC: distal colon cancer; RC: rectal cancer. *P*: test for overall effect. *P*_h_: value for heterogeneity. Bold indicates statistically significant P<0.05.

#### Heterogeneity

There was significant heterogeneity for case-control studies (*P*<0.01, *I*^*2*^=79%) and low heterogeneity between cohort studies (*P*=0.17, *I*^*2*^=26%) (Table 1). Subgroup analyses of cohort studies were conducted (Supplementary Table 1) to further identify the potential sources of heterogeneity and showed that the differences in RRs were not significant (*P*>0.05) for geographic area, sample size, publication year, quality score, smoking, alcohol, BMI, energy intake but were significant for physical activity and dietary fiber intake.

#### Publication bias

A funnel plot, Begg’s test and Egger’s test were used to assess publication bias. The funnel plot (Supplementary Figure 3), Egger’s test (*P*=0.23) and Begg’s test (*P*=0.75) did not indicate publication bias for cohort studies. Additionally, sensitivity analyses of cohort studies showed that the changes in recalculated RRs were not significant, with a range from 1.10 (1.01-1.19) when excluding Larsson 2006 (7.9%) to 1.15 (1.06-1.24) when excluding Ollberding 2012 (17.7%).

#### Dose-response analysis

Nine cohort studies were included, and the pooled RR was 1.16 (1.05-1.29) without heterogeneity (*P*=0.60, *I*^*2*^=0%) for 100 g/day increase. The results demonstrated a significant positive association between red meat intake and CRC risk. Sensitivity analysis also showed that the changes in recalculated RRs were not significant, with a range from 1.14 (1.01-1.29) when excluding Norat 2005 (33.9%) to 1.19 (1.07-1.32) when excluding Lee 2009 (4.9%). Additionally, non-linear associations were explored and the analysis did not suggest significant evidence of non-linear dose-response between processed meat consumption and CRC (*P*_for nonlinearity_=0.97).

#### Red meat-CC (PCC, DCC)

Twenty-six studies (15 case-control studies and 11 cohort studies) showed results for high vs low consumption of red meat and CC risk. The pooled RRs were 1.26 (1.10-1.43) for case-control studies (Supplementary Figure 1B) and 1.12 (1.02-1.20) for cohort studies (Figure 2B). The subgroup analysis of the cohort studies (Supplementary Table 2) suggested that the differences in RRs were significant for sample size and dietary fiber. Additionally, eight cohort studies were included in the dose-response analysis, and the result was 1.10 (0.96-1.26) without heterogeneity (*P*=0.82, *I*^*2*^=0%), which suggested that a 100 g/day increase in red meat consumption is not associated with a significant increase in CC risk (*P*=0.19). Sensitivity analysis of dose-response analysis showed that the changes in recalculated RRs were not significant, with a range from 1.04 (0.87-1.24) when excluding Norat 2005 (38.0%) to 1.11 (0.97-1.28) when excluding Lee 2009 (4.7%). Subtype analyses of cohort studies (Table 1) showed that red meat consumption was associated with PCC risk (RR=1.11, 95% CI=1.02-1.20). By contrast, the results of 1.36 (0.87-2.14) did not support a significant association between red meat consumption and DCC risk.

#### Red meat-RC

Twenty studies (12 case-control studies and 8 cohort studies) reported data for high vs low consumption of red meat and RC risk. The pooled RRs showed significant results, with 1.30 (1.10-1.52) for case-control studies (Supplementary Figure 1E) but null results for cohort studies (RR=1.12, 95% CI=0.89-1.40) (SupplementaryFigure 1E). Furthermore, subgroup analysis of the cohort studies (Supplementary Table 3) suggested that the results of each of the subgroup analyses were consistent for overall pooled estimates. Six cohort studies were included, and the pooled RR was 1.22 (1.01 to 1.47) without heterogeneity (*P*=0.66, *I*^*2*^=0%) for 100 g/day increase. However, sensitivity analysis showed that the changes in recalculated RRs were significant, with a range from 1.20 (0.93-1.56) when excluding Larsson 2005 (48.3%) to 1.25 (1.03-1.52) when excluding Lee 2009 (5.4%).

### Processed meat-CRC

#### High vs low consumption

**Figure 3:**
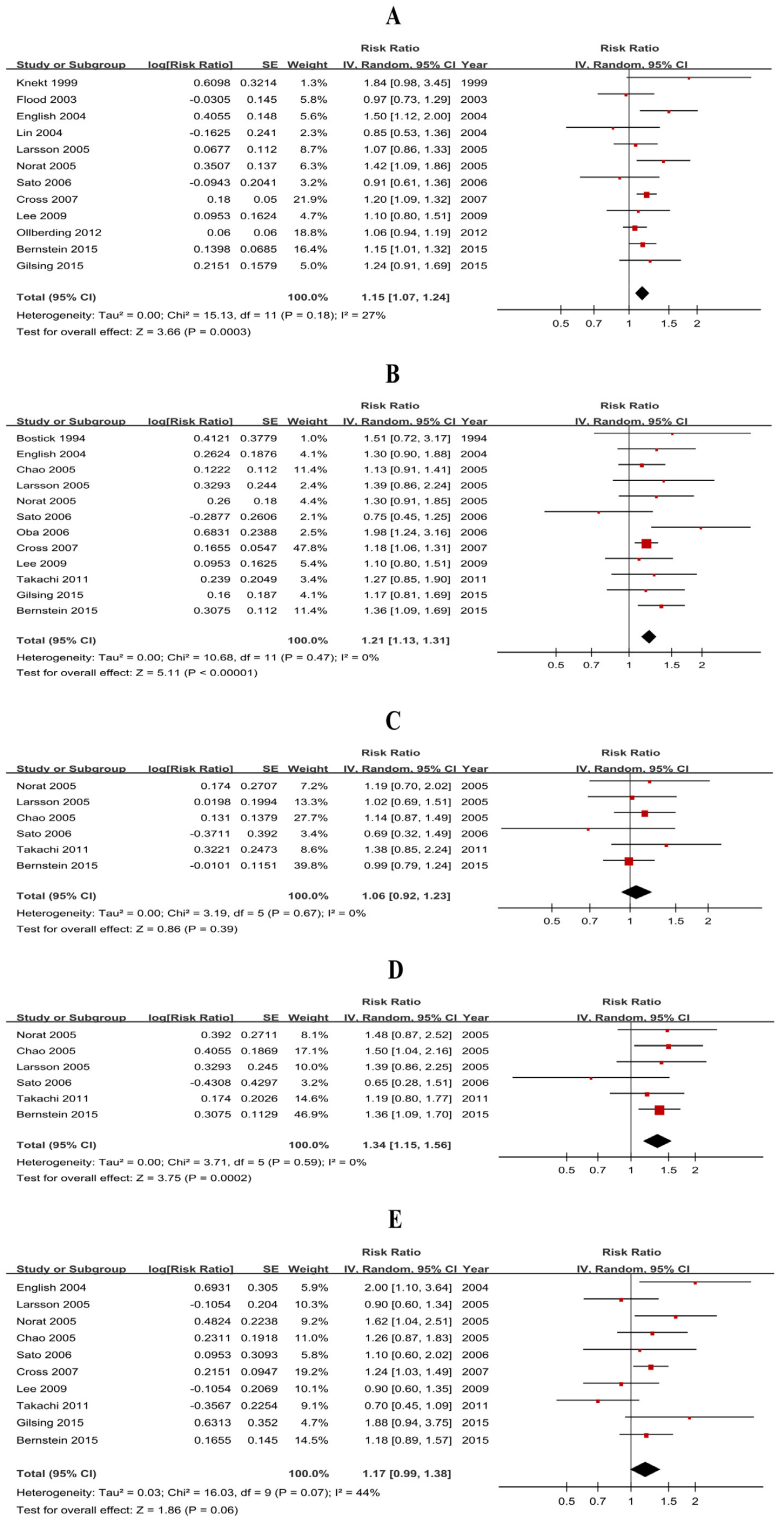
Forest plots of cohort studies for processed meat consumption (highest vs lowest) and colorectal cancer risk **(A)** Colorectal cancer; **(B)** colon cancer; **(C)** proximal colon cancer; **(D)** distal colon cancer; **(E)** rectal cancer.

Twenty-three studies (11 case-control studies and 12 cohort studies) were included and the pooled RRs were 1.36 (1.09-1.69) for case-control studies (Supplementary Figure 2A) and 1.15 (1.07-1.24) for cohort studies (Figure 3A).

#### Heterogeneity

There was significant heterogeneity (*P*<0.01, *I*^*2*^=76%) for case-control studies and low heterogeneity (*P*=0.18, *I*^*2*^=27%) between cohort studies. Subgroup analyses of cohort studies were conducted to further identify the potential sources of heterogeneity and suggested that the differences in RRs were not significant (*P*>0.05) between all subgroups (Supplementary Table 1).

#### Publication bias

A funnel plot, Begg’s test and Egger’s test were used to assess publication bias. The funnel plot (Supplementary Figure 4), Egger’s test (*P*=0.71) and Begg’s test (*P*=0.73) did not suggest significant evidence for publication bias for cohort studies. Sensitivity analyses of cohort studies also suggested that the changes in recalculated RRs were not significant, with a range from 1.14 (1.04-1.25) when excluding Cross 2007 (7.9%) to 1.17 (1.08-1.27) when excluding Ollberding 2012 (7.7%).

#### Dose-response analysis

Eight cohort studies were included, and the pooled RR was 1.22 (1.12-1.33) with low heterogeneity (*P*=0.28, *I*^*2*^=19%) for 50 g/day increase. The results showed a significant positive association between red meat intake and CRC risk. Sensitivity analysis also showed that the changes in recalculated RRs were not significant, with a range from 1.21 (1.07-1.37) when excluding Cross 2007 (33.6%) to 1.25 (1.12-1.40) when excluding Norat 2005 (33.2%). Non-linear association analysis suggested there was no significant evidence of non-linear dose-response between processed meat consumption and CRC (*P*_for nonlinearity_=0.54).

#### Processed meat-CC (PCC, DCC)

Twenty-three studies (11 case-control studies and 12 cohort studies) were included and the pooled RRs were 1.33 (1.17-1.51) for case-control studies (Supplementary Figure 2) and 1.21 (1.13-1.31) for cohort studies (Figure 3). The subgroup analysis of the cohort studies suggested that the results of each of the subgroup analyses were consistent for overall pooled estimates (Supplementary Table 2). Eight cohort studies were included in dose-response analysis, and the result was 1.23 (1.11 to 1.37) without heterogeneity (*P*=0.52, *I*^*2*^=0%), which suggested that a 50 g/day increase in processed meat consumption was not associated with a significant increase in CC risk. Sensitivity analysis of dose-response analysis showed that the changes in recalculated RRs were not significant, with a range from 1.22 (1.10-1.35) when excluding English 2004 (5.0%) to 1.31 (1.12-1.53) when excluding Norat 2005 (56.4%). Subtype analyses of cohort studies (Table 1) showed that red meat consumption was associated with DCC risk (RR=1.34, 95% CI=1.15-1.56). By contrast, the results of 1.06 (0.92-1.23) did not support a significant association between processed meat consumption and PCC risk.

#### Processed meat-RC

Nineteen studies (9 case-control studies and 10 cohort studies) were included and the pooled RRs were 1.28 (1.01-1.64) for case-control studies (Supplementary Figure 2E) but null results (RR=1.17, 95% CI=0.99-1.38) for cohort studies (Figure 3E). Furthermore, subgroup analysis of cohort studies (Supplementary Table 3) suggested that the results of each of the subgroup analyses were similar to overall pooled estimates (Supplementary Table 2). Seven cohort studies were included in dose-response analysis, and the pooled RR of 1.22 (0.99 to 1.28) was also not significant, without heterogeneity (*P*=0.41, *I*^*2*^=2%) for 50 g/day increase.

## DISCUSSION

Our findings provide detailed evidence that positive associations could be observed for CRC. However, the pooled estimate and the separate estimates of case-control and cohort studies for red meat consumption were negative for PCC. The pooled estimate and the separate estimates of case-control and cohort studies for processed meat consumption were negative for DCC. Additionally, there were no significant associations between red meat and processed meat consumption and the risk for RC in cohort studies. Overall, our detailed findings further clarify the associations between red and processed meat consumption and the risk of CRC types. These provide valuable detail to allow updating of the dietary recommendations.

Several potential mechanisms may contribute to the effects. First, the positive associations in the case-control studies may be biologically plausible. The cooking of meat is one of the major sources of carcinogens such as polycyclic aromatic hydrocarbons, heterocyclic amines, nitrate and *N*-nitroso compounds, which are believed to play important roles in the development of CRC [6]. Furthermore, the high iron intake associated with red and processed meat consumption may also play a role in CRC by causing oxidative damage and involving the endogenous formation of carcinogenic *N*-nitroso compounds [7]. Positive associations have been reported to be due to genetic differences. Specific genetic polymorphisms [8], xenobiotic metabolizing genes [9] and genetic susceptibility [10] have all been implicated in the pathogenesis of CRC. Finally, colorectal adenomashave been deemed to be a significant risk factor of CRC [11]. Studies [12, 13] have shown that high consumption of red and processed meat is associated with elevated colorectal adenomas.Nevertheless, the results of some cohort studies and meta-analyses do not support these explanations. For example, a multiethnic prospective investigation into cancer and nutrition suggested no potential association between higher consumption of red and processed meat and the risk of CRC [14]. Although some prospective studies have shown positive associations between red meat consumption and gastrointestinal cancer, the definition of red meat in these studies included processed red meat, which may have contributed to the positive association of cancer with red meat consumption [15]. Thus, further studies are needed to verify these potential mechanisms.

### Study strengths and limitations

Our study has several strengths. We performed separate analyses based on the study design and the locations of CRC. These independent analyses provided more detailed data and increased the power of the meta-analysis, which further strengthened the conclusion. We broadly and systematically reviewed databases for all investigations of red and processed meat consumption and the risk of CRC from the time of database inception through September 2016, which allowed us to identify all major published studies. Study selection and data extraction were performed independently and in duplicate by two investigators, thereby increasing the validity of the results. Additionally, studies were identified from 20 countries or regions in the Americas, Europe, Asia, and Australia, which increased the general applicability of the results. Our analysis is based on a substantial sample size and a quantitative synthesis of the eligible data (Supplementary Table 4). These data provided sufficient reliable, robust and current evidence and increased the statistical power of the analysis. Furthermore, dose-response analyses were conducted to assess these associations rather than simply performing categorical comparisons.

However, the limitations of the present meta-analysis must be taken into consideration. First, the included studies were observational, and residual confounding and unmeasured factors cannot be excluded. Nevertheless, most included studies were adjusted for potential confounders, including sex, age, body mass index (BMI), energy intake, physical activity, alcohol use and smoking. Furthermore, we performed subgroup analyses based on the main adjustment for confounders including smoking status, alcohol use, BMI, energy intake, physical activity and dietary fiber intake to evaluate the effects of these confounders. Generally, our findings were similar to the overall pooled estimates and were consistent for each of the subgroup analyses. However, information on some of the major confounders could not be obtained from some of the studies. In particular, most of the included studies lacked information concerning colorectal adenomas. Only two studies [14, 16] examined whether the association was modified by colorectal adenoma. Thus, this aspect of the results should be considered with caution due to possible confounding effects.

Second, our analyses showed significant heterogeneity among the studies, which may be related to the publication year, number of cases, geographic region, method of exposure measurement, quality score of the study, classification of meat consumption, and other confounders. We performed subgroup analyses to explore sources of heterogeneity and to avoid the influence of confounders. The range from the lowest to highest categories varied, and the consumption levels of red and processed meat between the lowest and highest categories differed between the included studies. Heterogeneity was observed mainly in the overall analysis comparing the highest vs the lowest consumption, which, at least in part, can be explained by the different categories of meat consumption. We used random-effects models to account for heterogeneity. Our analyses documented positive associations in most of the case-control studies, which drove the stronger effect of the case-control studies compared with the cohort studies in most of the analyses. Nevertheless, many included case-control studies provided exposure information obtained after the cancer diagnosis, which may be subject to inaccurate measurement of dietary intake and recall bias. Thus, the results from retrospective studies should not be overemphasized, and the results of prospective studies may be more informative than retrospective studies.

Third, the quality of several of the included studies was not high despite meeting the eligibility criteria, and the sample size regarding our topic was not large [17, 18]. Nevertheless, the subgroup analyses addressed these issues.

Finally, our analysis did not perform a subgroup analysis of the types of red and processed meat, i.e., beef, pork, lamb, mutton, bacon, lunch meat, ham, sausage, hot dogs, smoked meat and salted meat. Our study did not investigate the associations of CRC risk with other dietary factors, such as white meat, cooking techniques and heme iron from meat.

## MATERIALS AND METHODS

### Selection criteria

The selection criteria were as follows: histological features that were not consistent with the diagnostic gold standard were excluded; data that were incomplete or could not be combined were excluded; systematic reviews, narrative reviews, meta-analyses, editorials, letters, comments, case reports and studies in which only the abstract could be obtained were excluded; white meats and total meats without citing red or processed meat consumption were excluded; colorectal polyps, adenomas and other colorectal tumors were excluded; the studies were limited to those involving humans; and the language of all studies was limited to English.

### Red meat and processed meat

According to the World Cancer Research Fund (WCRF, http://wcrf.org/int/research-we-fund/cancer-prevention-recommendations/animal-foods), red meat in this study included beef, pork, lamb, mutton, beef burgers, veal, horse, liver and others. Processed meat included bacon, bacon rashers, lunch meat, ham, sausage, salami, hot dogs, souse meat, smoked meat, salted meat and others.

### Search strategy

We searched PubMed and EMBASE for studies published from inception through September 2016. The following search terms were used: “meat/beef/veal/pork/lamb/mutton/bacon/ham/sausage/salami/hot dogs/diet/dietary/food/foods” in combination with “gastrointestinal/digestive/alimentary tract/colorectal/colon/colonic/rectal/large bowel”, “neoplasia/cancer/carcinoma/adenomas/adenocarcinoma”. The reference lists of the included studies were also searched manually to identify additional literature. The two sets of keywords were combined individually, and the eligibility criteria were independently judged by two authors (ZZ and ZY).

### Data extraction and study quality

A data extraction sheet was generated for each study and included the first author, year of publication, country, study type, study population, study period, method of dietary assessment, type of dietary exposure measured, dietary exposure categories, adjusted RR (95% CI) (highest to lowest), adjusted variables and Newcastle-Ottawa Scale (NOS) score. Study quality in this meta-analysis was assessed using the NOS score, which is judged on three factors including the elucidation of the exposure or outcomes of interest for case-control or cohort studies, the selection of the study populations and the comparability of the populations [19]. Two authors (ZZ and ZY) independently assessed the quality of the studies, and discrepancies in interpretation were resolved by a consensus decision made by the third researcher (QZ). The range of NOS is 0-9 stars, and a study is considered high quality if it scores 7 or more stars.

### Statistical analysis

STATA version 12.1 (STATA Corporation, College Station, TX) and RevMan5.3 (The Cochrane Collaboration, Oxford, UK) software were used for data synthesis and analysis.

Random-effects models were used to pool the summary RRs and 95% confidence intervals (95% CI). The median or mean level of meat intake for each category was assigned to each corresponding RR for each study. When the data were not reported, the midpoint of the upper and lower boundaries in each category was assigned as the average intake. If the lowest category was open-ended, we assumed the lowest boundary to be 0. When the highest category was open-ended, we assumed the open-ended interval to be the same as that of the adjacent interval.

Heterogeneity among studies was detected using Q (a *P*<0.1 was considered representative of statistically significant heterogeneity) and *I*^2^ statistics (*I*^2^<50% was considered low heterogeneity and *I*^2^>50% was considered to indicate substantial heterogeneity) [20]. Subgroup analyses were conducted to further explore the sources of heterogeneity by geographic area, sample size, publication year, quality score, questionnaires used and adjustments (smoking, alcohol, BMI, energy intake, physical activity and dietary fiber intake).

Publication bias was assessed using funnel plots, Begg’s test and Egger’s test (*P*<0.1 was considered significant publication bias) [21]. Sensitivity analyses were conducted to investigate the influence of a specific study on the pooled risk estimate by removing one study in each turn. Nested case-control studies were included in the cohort studies.

## CONCLUSIONS

In a systematic review and meta-analysis, we found consumption of red and processed meat was associated with the risk of overall CRC but not RC. Additionally, there were no associations between the consumption of red meat and DCC risk and between the consumption of processed meat and PCC risk. Overall, our findings further clarify the associations between red meat and processed meat consumption and the risk of CRC, which can be used as a reference to update dietary recommendations.

## SUPPLEMENTARY MATERIALS FIGURES AND TABLES




