# Supplementary material for: Red and processed meat consumption and colorectal cancer risk: a systematic review and meta-analysis
Source: Oncotarget. 2017 Sep 6;8(47):83306–14. doi: 10.18632/oncotarget.20667 (PMC5669970; doi:10.18632/oncotarget.20667)
Supplement: Supplementary file 1 [file oncotarget-08-83306-s001.pdf]

Red and processed meat consumption and colorectal cancer risk: a systematic review and meta-analysis

SUPPLEMENTARY MATERIALS

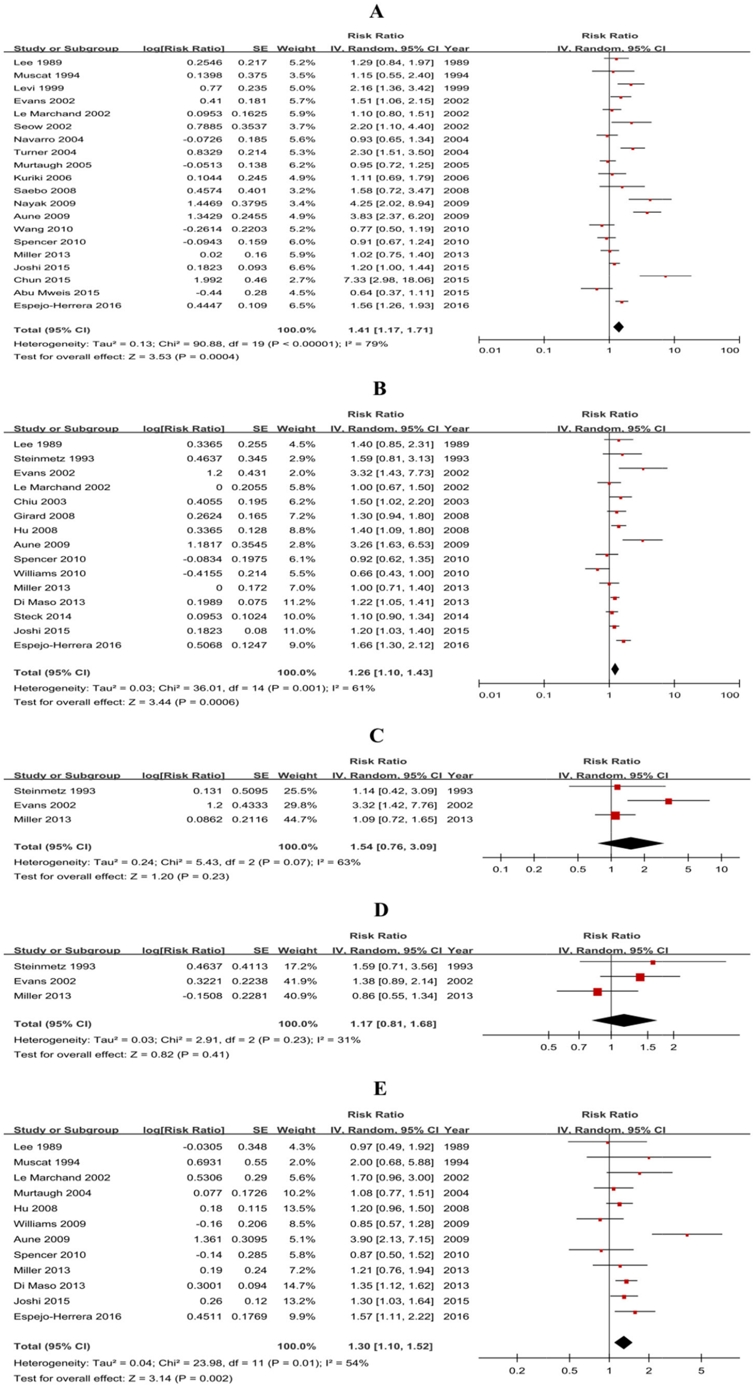

Supplementary Figure 1: Forest plots of case-control studies for red meat consumption (highest vs lowest categories) and colorectal cancer risk. **(A)** Colorectal cancer; **(B)** colon cancer; **(C)** proximal colon cancer; **(D)** distal colon cancer; **(E)** rectal cancer.

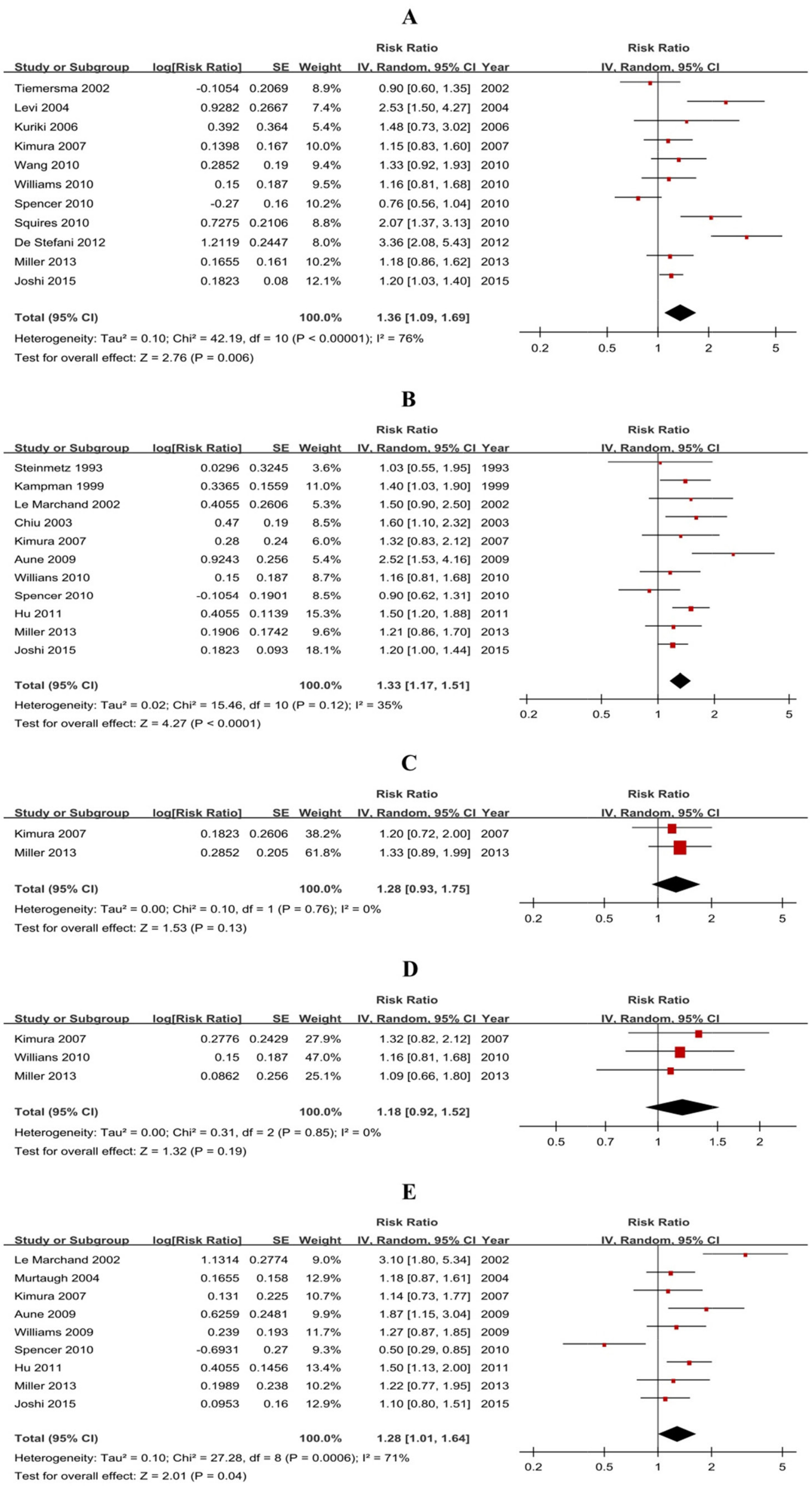

Supplementary Figure 2: Forest plots of case-control studies for processed meat consumption (highest vs lowest categories) and colorectal cancer risk. **(A)** Colorectal cancer; **(B)** colon cancer; **(C)** proximal colon cancer; **(D)** distal colon cancer; **(E)** rectal cancer.

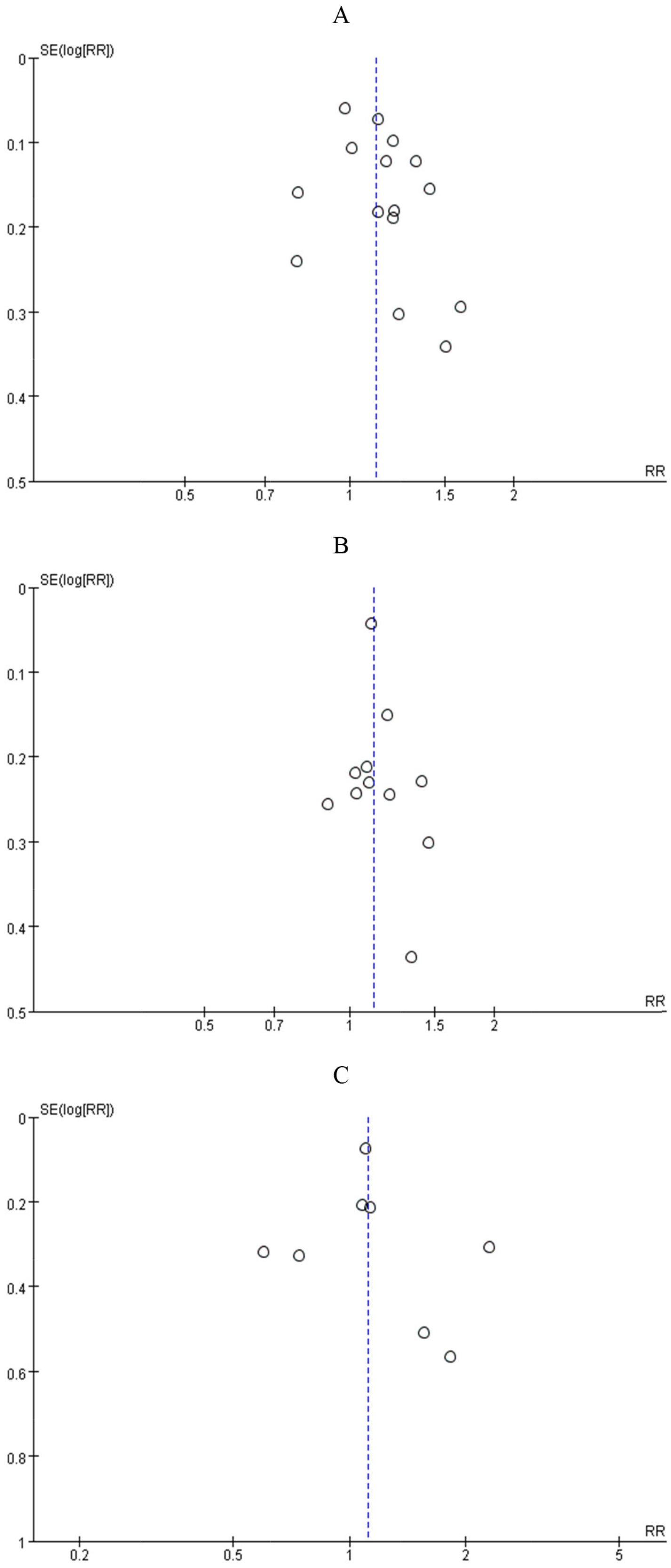

Supplementary Figure 3: Funnel plots evaluating publication bias of cohort studies of red meat consumption and colorectal cancer risk. **(A)** Colorectal cancer; **(B)** colon cancer; **(C)** rectal cancer.

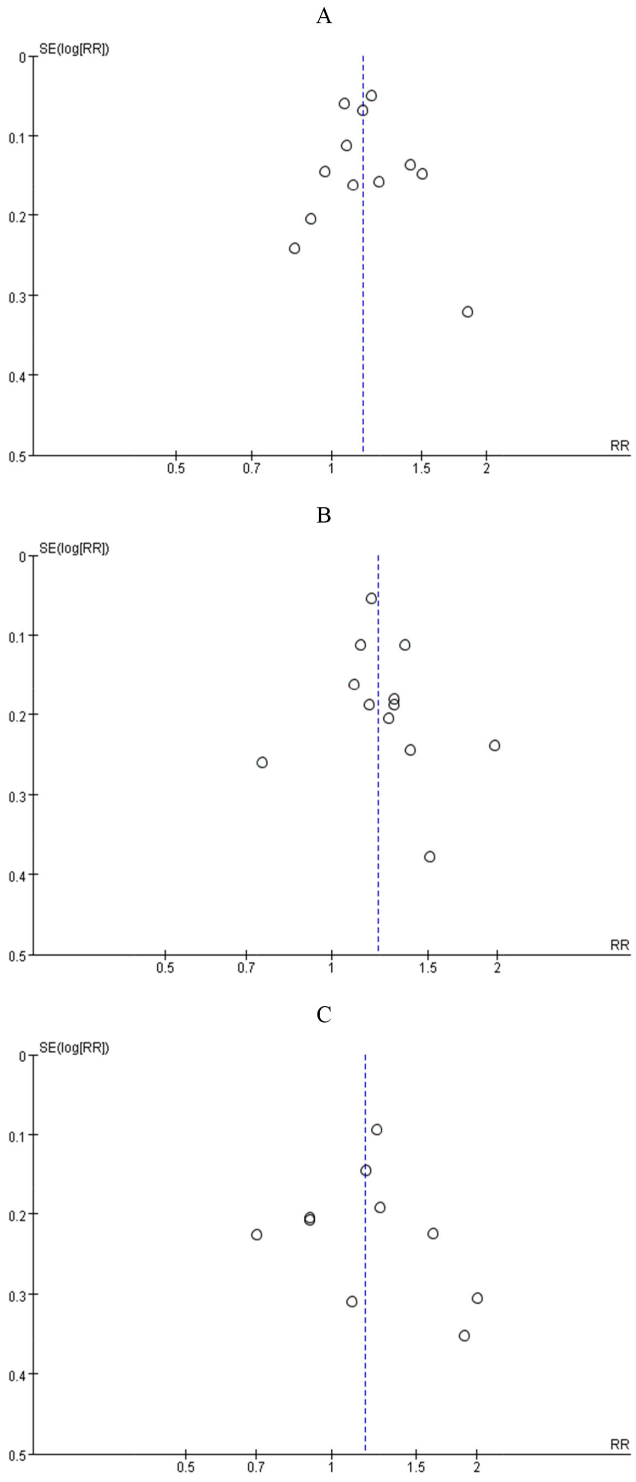

Supplementary Figure 4: Funnel plots evaluating publication bias of cohort studies of processed meat consumption and colorectal cancer risk. **(A)** Colorectal cancer; **(B)** colon cancer; **(C)** rectal cancer.

Supplementary Table 1: Subgroup analyses of cohort studies for red and processed meat consumption (highest vs lowest categories) and colorectal cancer risk

Subgroups	Red meat						Processed meat					
	N	RR (95% CI)	P_s	I_s^2 (%)	P_h	I_h^2 (%)	N	RR (95% CI)	P_s	I_s^2 (%)	P_h	I_h^2 (%)
**All studies**	16	**1.10 (1.02-1.19)**	.18	24			12	**1.15 (1.07-1.24)**	.18	27		
**Geographic area**												
**Europe**	6	**1.22 (1.06-1.40)**	.43	0			4	**1.26 (1.05-1.51)**	.24	29		
**America**	6	1.07 (0.99-1.16)	.38	6			5	**1.12 (1.04-1.21)**	.29	20		
**Asia-Australia**	4	1.06 (0.85-1.31)	.09	54	.26	24.8	3	1.17 (0.88-1.56)	.11	55	.50	0
**Sample size**												
**≥200**	11	**1.10 (1.01-1.19)**	.13	34			6	**1.14 (1.06-1.23)**	.26	23		
**<200**	5	1.18 (0.94-1.49)	.37	6	.54	0	6	1.18 (0.98-1.42)	.13	41	.75	0
**Publication year**												
**2005 or later**	11	**1.08 (1.00-1.17)**	.24	21			8	**1.15 (1.08-1.21)**	.45	0		
**Before 2005**	5	1.26 (0.99-1.60)	.29	20	.24	26.5	4	1.19 (0.87-1.65)	.04	64	.80	0
**Quality score**												
**≥7 stars**	14	**1.09 (1.01-1.18)**	.18	25			9	**1.14 (1.07-1.21)**	.40	4		
**<7 stars**	2	1.31 (0.97-1.76)	.42	0	.26	21.6	3	1.32 (0.89-1.94)	.08	61	.46	0
**Smoking**												
**Yes**	10	**1.06 (1.00-1.14)**	.49	0			9	**1.14 (1.05-1.24)**	.18	30		
**No**	6	1.21 (0.99-1.47)	.11	44	.24	28.1	3	1.20 (0.97-1.48)	.17	44	.67	0
**Alcohol**												
**Yes**	10	**1.09 (1.00-1.19)**	.23	23			9	**1.13 (1.05-1.21)**	.31	15		
**No**	6	1.14 (0.97-1.34)	.19	33	.62	0	3	1.36 (1.05-1.78)	.22	34	.17	46
**BMI**												
**Yes**	14	**1.12 (1.04-1.20)**	.26	18			9	**1.13 (1.05-1.21)**	.31	15		
**No**	2	0.92 (0.62-1.36)	.21	36	.34	0	3	1.36 (1.05-1.78)	.22	34	.17	46
**Energy intake**												
**Yes**	12	**1.13 (1.03-1.23)**	.11	35			11	**1.16 (1.07-1.25)**	.18	28		
**No**	4	1.03 (0.87-1.21)	.49	0	.34	0	1	0.91 (0.61-1.36)	-	-	.25	25.7
**Physical activity**												
**Yes**	7	1.04 (0.96-1.13)	.40	3			8	**1.13 (1.05-1.22)**	.24	24		
**No**	9	1.18 (1.05-1.33)	.27	19	.08	67.6	4	1.25 (1.01-1.55)	.15	44	.40	0
**Dietary fiber**												
**Yes**	10	1.09 (0.99-1.19)	.11	38			4	**1.15 (1.06-1.25)**	.47	0		
**No**	6	1.18 (1.03-1.36)	.58	0	.32	0	8	1.18 (1.05-1.33)	.08	44	.73	0

BMI: body mass index. P_s: P value for heterogeneity within each subgroup. P_h: P value for heterogeneity between subgroups. I_s^2: I^2 value for heterogeneity within each subgroup. I_h^2: I^2 value for heterogeneity between subgroups. Bold text indicates statistical significance.

Supplementary Table 2: Subgroup analyses of cohort studies for red and processed meat consumption (highest vs lowest categories) and colon cancer risk

Subgroups	Red meat						Processed meat					
	n	RR (95% CI)	P_s	I_s^2 (%)	P_h	I_h^2 (%)	n	RR (95% CI)	P_s	I_s^2 (%)	P_h	I_h^2 (%)
**All studies**	11	**1.12 (1.04-1.20)**	.97	0			12	**1.15 (1.07-1.24)**	.18	27		
**Geographic area**												
**Europe**	4	1.14 (0.93-1.40)	.91	0			3	**1.27 (1.01-1.59)**	.85	0		
**America**	3	**1.12 (1.03-1.22)**	.56	0			4	**1.20 (1.10-1.31)**	.57	0		
**Asia-Australia**	4	1.08 (0.84-1.39)	.67	0	.95	0	5	1.23 (0.95-1.59)	.09	51	.91	0
**Sample size**												
**≥200**	8	1.12 (0.96-1.30)	.95	0			9	**1.24 (1.11-1.38)**	.26	21		
**<200**	3	1.18 (1.03-1.22)	.54	0	.97	0	3	1.18 (0.96-1.44)	.80	0	.68	0
**Publication year**												
**2005 or later**	7	**1.11 (1.03-1.20)**	.92	0			10	**1.21 (1.11-1.33)**	.34	12		
**Before 2005**	4	1.24 (0.97-1.60)	.89	0	.97	0	2	1.34 (0.96-1.86)	.72	0	.57	0
**Quality score**												
**≥7 stars**	11	**1.12 (1.04-1.20)**	.97	0			10	**1.22 (1.13-1.32)**	.63	0		
**<7 stars**	0	-	-	-	-	-	2	1.02 (0.60-1.74)	.09	66	.51	0
**Smoking**												
**Yes**	7	**1.13 (1.04-1.22)**	.89	0			8	**1.22 (1.09-1.37)**	.22	27		
**No**	4	1.06 (0.84-1.33)	.86	0	.61	0	4	1.24 (1.01-1.53)	.78	0	.90	0
**Alcohol**												
**Yes**	7	**1.12 (1.04-1.21)**	.89	0			9	**1.23 (1.11-1.36)**	.28	19		
**No**	4	1.09 (0.84-1.42)	.81	0	.84	0	3	1.21 (1.11-1.36)	.66	0	.91	0
**BMI**												
**Yes**	8	**1.18 (1.01-1.39)**	.96	0			10	**1.23 (1.12-1.35)**	.34	12		
**No**	3	1.10 (1.02-1.20)	.72	0	.43	0	2	1.18 (0.93-1.50)	.50	0	.77	0
**Energy intake**												
**Yes**	8	**1.11 (1.03-1.20)**	.99	0			10	**1.21 (1.12-1.31)**	.96	0		
**No**	3	1.27 (0.95-1.69)	.56	0	.37	0	2	0.91 (0.61-1.36)	<.01	87	.98	0
**Physical activity**												
**Yes**	6	**1.13 (1.04-1.22)**	.83	0			8	**1.22 (1.09-1.37)**	.22	27		
**No**	5	1.07 (0.86-1.34)	.91	0	.70	0	4	1.24 (1.01-1.53)	.78	0	.90	0
**Dietary fiber**												
**Yes**	5	1.14 (0.93-1.39)	.74	0			5	**1.18 (1.08-1.28)**	.92	0		
**No**	6	1.12 (1.03-1.21)	.94	0	.85	0	7	1.30 (1.10-1.54)	.23	27	.31	1.6

BMI: body mass index. P_s: P value for heterogeneity within each subgroup. P_h: P value for heterogeneity between subgroups. I_s^2: I^2 value for heterogeneity within each subgroup. I_h^2: I^2 value for heterogeneity between subgroups. Bold text indicates statistical significance.

Supplementary Table 3: Subgroup analyses of cohort studies for red and processed meat consumption (highest vs lowest categories) and rectal cancer risk

Subgroups	Red meat						Processed meat					
	n	RR (95% CI)	P_s	I_s^2 (%)	P_h	I_h^2 (%)	n	RR (95% CI)	P_s	I_s^2 (%)	P_h	I_h^2 (%)
**All studies**	8	1.12 (0.89-1.40)	.10	42			10	1.17 (0.99-1.38)	.07	44		
**Geographic area**												
**Europe**	4	1.10 (0.95-1.27)	.78	0			3	**1.23 (1.06-1.42)**	.95	0		
**America**	3	1.01 (0.44-2.33)	-	-			3	1.34 (0.84-2.13)	.07	62		
**Asia-Australia**	1	1.17 (0.89-1.53)	<.01	81	.91	0	4	1.04 (0.69-1.57)	.05	63	.70	0
**Sample size**												
**≥200**	3	1.10 (0.97-1.25)	.99	0			7	1.14 (0.96-1.35)	.15	37		
**<200**	5	1.19 (0.66-2.13)	.02	67	.81	0	3	1.43 (0.81-2.53)	.04	68	.44	0
**Publication year**												
**2005 or later**	6	1.05 (0.91-1.22)	.35	8			9	1.13 (0.97-1.33)	.12	38		
**Before 2005**	2	2.18 (1.28-3.70)	.72	0	<.01	85.2	1	2.00 (1.10-3.64)	-	-	.07	69
**Quality score**												
**≥7 stars**	8	1.12 (0.89-1.40)	.10	42			8	1.13 (0.95-1.35)	.08	46		
**<7 stars**	0	-	-	-	-	-	2	1.49 (0.83-2.67)	.17	47	.39	0
**Smoking**												
**Yes**	5	1.10 (0.96-1.26)	.60	0			7	**1.21 (1.01-1.44)**	.15	36		
**No**	3	1.14 (0.58-2.24)	<.01	79	.91	0	3	1.11 (0.72-1.74)	.06	64	.75	0
**Alcohol**												
**Yes**	5	1.09 (0.96-1.24)	.75	0			8	1.16 (0.98-1.38)	.12	38		
**No**	3	1.33 (0.52-3.41)	<.01	79	.69	0	2	1.30 (0.60-2.84)	.03	79	.79	0
**BMI**												
**Yes**	5	1.09 (0.85-1.40)	.60	0			8	1.16 (0.98-1.38)	.12	38		
**No**	3	1.15 (0.65-2.03)	<.01	79	.87	0	2	1.30 (0.60-2.84)	.03	79	.79	0
**Energy intake**												
**Yes**	6	1.22 (0.85-1.75)	.07	51			9	1.18 (0.98-1.41)	.04	50		
**No**	2	1.03 (0.76-1.38)	.24	28	.47	0	1	1.10 (0.60-2.02)	-	-	.83	0
**Physical activity**												
**Yes**	4	1.09 (0.95-1.25)	.58	0			7	**1.21 (1.01-1.44)**	.15	36		
**No**	4	1.23 (0.69-2.18)	.02	70	.69	0	3	1.11 (0.72-1.74)	.06	64	.75	0
**Dietary fiber**												
**Yes**	6	1.11 (0.77-1.58)	.04	57			5	1.02 (0.82-1.27)	.09	50		
**No**	2	1.11 (0.96-1.28)	.50	0	.99	0	5	1.40 (1.12-1.74)	.35	10	.05	75

BMI: body mass index. P_s: P value for heterogeneity within each subgroup. P_h: P value for heterogeneity between subgroups. I_s^2: I^2 value for heterogeneity within each subgroup. I_h^2: I^2 value for heterogeneity between subgroups. Bold text indicates statistical significance.

Supplementary Table 4: Baseline characteristics of included studies for red and processed meat consumption and colorectal cancer risk

See Supplementary File 1
