# Supplementary material for: Red and processed meat consumption and colorectal cancer risk: a systematic review and meta-analysis
Source: Oncotarget. 2017 Sep 6;8(47):83306–14. doi: 10.18632/oncotarget.20667 (PMC5669970; doi:10.18632/oncotarget.20667)
Supplement: Supplementary file 2 [file oncotarget-08-83306-s002.docx]

**Supplementary Table 4**. Baseline characteristics of included studies for red and processed meat consumption and colorectal cancer risk.

| **First author, year, country** | **Study**  **type** | **Case/control**  **(cohort, n)** | **Study period** | **Method of dietary assessment** | **Type of dietary exposure** | **Dietary exposure categories** | **Adjusted RRs/ORs (95% CI)**  **(highest to lowest)** | **Adjusted variables** | **NOS score** |
| --- | --- | --- | --- | --- | --- | --- | --- | --- | --- |
| Lee 1989 Singapore[1] | cc | Colorectal cancer:  203/425  Colon cancer:  132/425  Rectal cancer:  71/425 | 1985-1987 | FFQ-116 | Pork, beef, mutton | High vs med | Colorectal cancer:  1.29 (0.84-1.97)  Colon cancer:  1.41 (0.87-2.31)  Rectal cancer:  0.97 (0.48-1.92) | age, sex, dialect group and occupation | 7 |
| Steinmetz 1993 USA[2] | cc | Colon cancer:  220/438 | 1979-1980 | FFQ-141 | Red meat | Quartile | Colon cancer:  RD: 1.59 (0.81-3.13)  PD: 1.03 (0.55-1.95) | occupation, Quetelet's index and alcohol intake | 6 |
| Muscat 1994 USA[3] | cc | Colorectal cancer:  511/500  Rectal cancer:  165/500 | 1989-1992 | FFQ-NS | Beef | Tertile | Colorectal cancer:  1.15 (0.60-2.40)  Rectal cancer:  2.00 (0.70-5.90) | sex, race, hospital, age, time of the case interview | 7 |
| Levi 1999 Switzerland[4] | cc | 223/491 | 1992-1997 | FFQ-79 | Red meat | Tertile | 2.15 (1.35-3.42) | age, sex, education, smoking, alcohol, BMI, physical activity and total energy intake | 7 |
| Kampman 1999 USA[5] | cc | Colon cancer  1542/1860 | 1992-1995 | FFQ-NS | Processed meat | Quintile | 1.40 (1.03-1.90) | Age, calories, BMI, long-term activity, dietary fiber, smoking | 7 |
| Evans 2002 Italy[6] | cc | Colorectal cancer:  512/512  Colon cancer:  112/512 | 2002  (6 months) | FFQ-160 | Red meat | Quartile | Colorectal cancer:  1.51 (1.06-2.15)  Colon cancer:  3.32 (1.42-7.73) | no | 6 |
| Le Marchand 2002 USA[7] | cc | Colorectal cancer:  723/727  Colon cancer:  513/727  Rectal cancer:  210/727 | 1994-1998 | FFQ-NS | Red meat  Processed meat | Tertile | Colorectal cancer:  1.10 (0.80-1.60)  Colon cancer:  RD: 1.00 (0.70-1.50)  PD: 1.50 (0.90-2.50)  Rectal cancer:  RD: 1.70 (1.00-3.00)  PD: 3.10 (1.80-5.34) | smoking, physical activity, aspirin use, BMI, education, non-starch polysaccharides from vegetables and total calcium | 8 |
| Seow 2002 Singapore[8] | cc | 121/222 | 1999-2000 | FFQ-NS | Red meat | Tertile | 2.20 (1.10-4.20) | age, family history of CRC, gender, smoking, education, and usual number of hours of moderate/vigorous exercise | 6 |
| Tiemersma 2002 Netherlands[9] | cc | 102/537 | 1993-1998 | FFQ-NS | Red meat  Sausage | Tertile | 1.60 (0.90-2.90)  1.00 (0.50-1.90) | age, sex, center, total energy intake, alcohol consumption, and BMI | 6 |
| Chiu 2003 China[10] | cc | Colon cancer:  931/1552 | 1990-1993 | FFQ-NS | Fresh red meat  Processed meat | Quartile | Colon cancer:  1.50 (1.00-2.20)  1.60 (1.10-2.32)) | age, total energy, education, BMI, income and occupational physical activity | 7 |
| Levi 2004 Switzerland[11] | cc | 323/611 | 1992-2002 | FFQ-79 | Processed meat | Quartile | 2.53 (1.50-4.27) | education, smoking, drinking, energy intake, fruit and vegetable | 7 |
| Murtaugh 2004 USA[12] | cc | Rectal cancer:  952/1025 | 1997-2002 | FFQ-NS | Red meat  Processed meat | Tertile | Rectal cancer:  1.08 (0.77-1.81)  1.18 (0.87-1.61) | age, BMI, energy intake, dietary fiber, calcium, lifetime physical activity, and usual number of cigarettes smoked | 7 |
| Navarro 2004 Argentina[13] | cc | 296/597 | 1994-2000 | FFQ-NS | Red meat | Tertile | 0.93 (0.65-1.34) | sex, age, BMI, smoking habit, and SES | 6 |
| Turner 2004 UK[14] | cc | 500/500 | 1997-2001 | FFQ-NS | Red meat | Quartile | 2.30 (1.60-3.50) | age, sex and general practitioner | 7 |
| Chan 2005 USA[15] | cc | 183/443 | 1976-1990 | FFQ-NS | Beef/veal/lamb | ≥0.5 vs <0.5 serving/d | 1.21 (0.85-1.72) | age, BMI, CRC in a parent or sibling, previous endoscopy, current multivitamin use and regular aspirin use, post-menopausal hormone use | 6 |
| Murtaugh 2005 USA[16] | cc | 2298/2749 | 1991-1994  1997-2002 | FFQ-NS | Red meat | Tertile | 0.95 (0.73-1.25) | age, BMI, energy intake, dietary fiber and calcium, lifetime physical activity, and smoking | 7 |
| Kuriki 2006 Japan[17] | cc | 257/771 | 1999-2000 | FFQ-47 | Beef/pork  Processed meat | Tertile | 1.11 (0.67-1.79)  1.48 (0.73-3.02) | BMI, habitual exercise, drinking, smoking, family history of CRC | 7 |
| Kimura 2007 Japan[18] | cc | Colorectal cancer:  782/793  Colon cancer:  262/262  Rectal cancer:  327/327 | 2000-2003 | FFQ-NS | Processed meat | Quintile | Colorectal cancer:  1.15 (0.83-1.60)  Colon cancer:  1.32 (0.83-2.12)  Rectal cancer:  1.14 (0.73-1.77) | age, sex, residential area, BMI, parental CRC, smoking, alcohol, job, leisure-time physical activity, dietary calcium and fiber | 7 |
| Girard 2008 USA[19] | cc | Colon cancer:  537/866 | 1996-2000 | FFQ-150 | Red meat | ≥28.5g/day vs <28.5g/day | Colon cancer:  1.30 (1.00-1.80) | age, race, sex, total meat, energy-adjusted fat intake, dietary fiber intake, total energy, and offsets | 7 |
| Hu 2008 Canada[20] | cc | Colon cancer:  1727/5039  Rectal cancer:  1447/5039 | 1994-1997 | FFQ-69 | Red meat | Quartile | Colon cancer:  1.40 (1.10-1.80)  Rectal cancer:  1.20 (1.00-1.50) | age, province, education, BMI, sex, alcohol use, smoking, total of vegetable and fruit intake, and total energy intake (adjusted for strenuous and moderate activity). | 8 |
| Saebo 2008 Norway[21] | cc | 198/222 | 1984-1997 | FFQ-NS | Red meat | Tertile | 1.58 (0.71-3.47) | age, gender, and in addition genotyping, diet parameters are adjusted for ever smoking | 6 |
| Aune 2009 Uruguay[22] | cc | Colorectal cancer:  361/2032  Colon cancer:  176/2032  Rectal cancer:  185/2032 | 1996-2004 | FFQ-64 | Red meat  Processed meat | Tertile | Colorectal cancer:  3.83 (2.37-6.20)  Colon cancer:  RD: 3.26 (1.63-6.53)  PD: 2.52 (1.53-4.16)  Rectal cancer:  RD: 3.90 (2.13-7.15)  PD: 1.87 (1.15-3.04) | age, sex, residence, education, income, interviewer, smoking, alcohol, dairy foods, grains, fatty foods, fruits and vegetables, fish, poultry, mate drinking, BMI and energy intake | 7 |
| Nayak 2009 India[23] | cc | 108/234 | 2003-2006 | FFQ-NS | Beef | Tertile | 4.25 (2.02-8.94) | age, sex, problem oriented medical history, socio-demographic characteristics and lifestyle habits (e.g. smoking and drinking etc.) | 6 |
| Williams 2009 USA[24] | cc | Rectal cancer:  720/800 | 2001-2006 | DHQ-124 | Red meat  Processed meat | Quartile | Rectal cancer:  0.85 (0.56-1.28)  1.27 (0.87-1.85) | age, sex, education, income. BMI, physical activity, family history, NSAID use, and total energy intake | 7 |
| Squires 2010 Canada[25] | cc | 518/686 | 1999-2003 | PHQ-NS, FFQ-NS | Pickled meat | Quartile | 2.51 (1.45-4.32) | age, BMI, smoking, education, vegetables, fruit, folic acid, cholesterol, dietary fiber, saturated fat, alcohol, caloric intake, physical activity, NSAID use, and the presence of IBD | 7 |
| Spencer 2010 UK[26] | cc | Colorectal cancer:  579/1996  Colon cancer:  380/1316  Rectal cancer:  199/680 | 1985-2003 | FFQ-NS | Red meat  Processed meat | Quartile | Colorectal cancer:  RD: 0.91 (0.66-1.24)  PD: 0.76 (0.56-1.03)  Colon cancer:  RD: 0.92 (0.62-1.35)  PD: 0.90 (0.62-1.31)  Rectal cancer:  RD: 0.87 (0.50-1.52)  PD: 0.50 (0.29-0.85) | age, BMI, smoking, intakes of energy, alcohol and dietary fibre | 7 |
| Wang 2010 USA[27] | cc | 496/607 | 1995-2007 | FFQ-200 | Red meat  Processed meat | Tertile | 0.77 (0.50-1.17)  1.33 (0.92-1.93) | age, sex and ethnicity, daily energy intake, physical activity, BMI, smoking, alcohol intake, folate intake | 7 |
| Williams 2010 USA[28] | cc | Colon cancer: 945/959 | 2001-2006 | DHQ-124 | Red meat  Processed meat | Quartile | Colon cancer:  RD: 0.66 (0.43-1.00)  PD :1.16 (0.80-1.68) | age, sex, education, BMI, family history, NSAID use, physical activity, calcium, fiber, total energy | 7 |
| Hu 2011 Canada[29] | cc | Colon cancer:  1727/5039  Rectal cancer:  1447/5039 | 1994-1997 | FFQ-69 | Processed meat | Quartile | Colon cancer:  1.50 (1.20-1.80)  Rectal cancer:  1.50 (1.20-2.0) | age, province, education, BMI, sex, alcohol, smoking, total vegetable and fruit, and total energy intake; adjusted for strenuous and moderate activity for colon and rectum cancer | 8 |
| De Stefani 2012 Uruguay[30] | cc | 234/2020 | 1996-2004 | FFQ-64 | Processed meat | Tertile | 4.57 (2.25-9.80) | age, gender, residence, education, BMI, smoking, drinking, mate temperature, total energy, total vegetable and fruits intake | 7 |
| Di Maso 2013 Italy[31] | cc | Colon cancer:  1463/4943  Rectal cancer:  927/4943 | 1991-2009 | FFQ-NS | Red meat | Tertile | Colon cancer:  1.22 (1.05-1.41)  Rectal cancer:  1.35 (1.12-1.62) | age, sex, education, BMI, smoking, alcohol, vegetable and fruit intake | 8 |
| Miller 2013 USA[32] | cc | Colorectal cancer:  989/1033  Colon cancer:  693/1033  Rectal cancer:  289/1033 | 2007-2011 | DHQ-NS, FFQ-137 | Unprocessed red meat  Processed meat | Quintile | Colorectal cancer:  RD: 1.02 (0.75-1.40)  PD: 1.18 (0.87-1.62)  Colon cancer:  RD: 1.00 (0.71-1.40)  PD: 1.21 (0.86-1.70)  Rectal cancer:  RD: 1.21 (0.76-1.94)  PD: 1.22 90.77-1.95） | age, sex, BMI, NSAID use, and intakes of total energy and fruits and vegetables | 8 |
| Steck 2014 USA[33] | cc | Colon cancer:  555/875 | 1996-2000 | FFQ-150 | Red meat | ≥28.4g/day vs <28.4g/day | Colon cancer:  1.10 (0.90-1.40) | age, race, gender, offsets, total energy intake, energy-adjusted fat intake, dietary fiber intake and total meat intake | 7 |
| Abu Mweis 2015 Jordan[34] | cc | 154/237 | 2010-2012 | FFQ-109 | Red meat | ≥1 vs <1 per week | 0.64 (0.37 -1.11) | age, sex, total energy, metabolic equivalent, smoking, education, marital status, work, income, and family history of CRC | 6 |
| Chun 2015 Korea[35] | cc | 150/116 | 2010-2011 | FFQ-NS | Red meat | Tertile | 7.33 (2.98-18.06) | energy intake, sex, age, income, education, smoking, alcohol, exercise, BMI, dietary fiber | 6 |
| Joshi 2015 USA[36] | cc | Colorectal cancer:  3504/3350  Colon cancer:  1992/3350  Rectal cancer:  907/3350 | 1997-2007 | RFQ, FFQ-NS | Red meat  Processed meat | Quintile | Colorectal cancer:  RD: 1.20 (1.00-1.40)  PD: 1.20 (1.10-1.40)  Colon cancer:  RD: 1.20 (1.00-1.50)  PD: 1.20 (1.00-1.44)  Rectal cancer:  RD: 1.30 (1.00-1.60)  PD: 1.10 (0.80-1.51) | age, BMI, gender, race, saturated fat, dietary fiber, vegetables, physical activity, total calorie intake | 8 |
| Espejo-Herrera 2016 Spain and Italy[37] | cc | Colorectal cancer:  1869/3530  Colon cancer:  1285/3530  Rectal cancer:  557/3530 | 2008-2013 | FFQ-140 | Red meat | >29 g/day vs ≤29 g/day | Colorectal cancer:  1.56 (1.26-1.94)  Colon cancer:  1.66 (1.30-2.12)  Rectal cancer:  1.57 (1.11-2.21) | age, sex, education, physical activity, NSAID use, family history of CRC and energy intake | 8 |
| Bostick 1994 USA[38] | co | Colon cancer:  212/35215 | 1986-1990 | FFQ-127 | Beef, lamb, or pork as main dish  Processed meat | Quartile | Colon cancer:  1.21 (0.75-1.96)  1.51 (0.72-3.17) | age, total energy intake, height, parity, Vitamin E and Vitamin A intake | 7 |
| Kato 1997 USA[39] | co | 100/14727 | 1985-1994 | FFQ-70 | Red meat | Quartile | 1.23 (0.68-2.22) | age, total calorie intake, place at enrollment and education | 7 |
| Singh 1998 USA[40] | co | Colon cancer:  157/178544 | 1976-1982 | FFQ-55 | Red meat | Tertile | Colon cancer:  1.41 (0.90-2.21) | age, sex, BMI, physical activity, parental history of CRC, smoking, alcohol consumption, aspirin use | 7 |
| Knekt 1999 Finland[41] | co | 73/9985 | 1966-1990 | Pre-formed questionnaire | Cured meat | Quartile | 1.84 (0.98-3.47) | age, sex, municipality, smoking and energy intake | 6 |
| Pietinen 1999 Finland[42] | co | 185/27111 | 1987-1995 | FFQ-276 | Beef, lamb, pork | Quartile | 0.80 (0.50-1.20) | age, smoking, BMI, alcohol, education, physical activity and calcium intake | 7 |
| Jarvinen 2001 Finland[43] | co | Colorectal cancer:  109/9959  Colon cancer:  63/9959  Rectal cancer:  46/9959 | 1972-1999 | FFQ, >100 | Red meat | Quartile | Colorectal cancer:  1.50 (0.77-2.94)  Colon cancer:  1.34 (0.57-3.15)  Rectal cancer:  1.82 (0.60-5.52) | age, sex, BMI, occupation, smoking, geographical area, energy intake, consumption of vegetables, fruits and cereals | 8 |
| Flood 2003 USA[44] | co | 487/45496 | 1987-1989  1992-1995  1995-1998 | FFQ-62 | Processed meat | Quintile | 1.00 (0.76-1.31) | age, sex, education, income, BMI, physical activity, family history, NSAID, total energy intake, smoking, height, alcohol, folate, vitamin D, calcium, fruits, vegetables, grains | 8 |
| Lin 2004 USA[45] | co | 202/37547 | 1993-2003 | FFQ-131 | Processed meat | Quintile | 0.85 (0.53-1.35) | age, random treatment assignment, BMI, family history of CRC and colorectal polyps, physical activity, smoking, alcohol, postmenopausal hormone therapy, energy intake | 7 |
| English 2004 Australia[46] | co | Colorectal cancer:  452/37112  Colon cancer:  283/37112  Rectal cancer:  169/37112 | 1990-1994 | FFQ-121 | Fresh red meat  Processed meat | Quartile | Colorectal cancer:  RD: 1.40 (1.00-1.90)  PD: 1.50 (1.10-2.00)  Colon cancer:  RD: 1.10 (0.70-1.60)  PD: 1.30 (0.90-1.88)  Rectal cancer:  RD: 2.30 (1.20-4.20)  PD: 2.00 (1.10-3.64) | sex, country of birth, intake of energy, fat, and cereal products using Cox’s proportional hazard model with age as the time metric | 8 |
| Chao 2005 USA[47] | co | Colon cancer:  1075/148610  Rectal cancer:  470/148610 | 1993-2001 | FFQ-68 | Processed meat | Quintile | Colon cancer:  1.13 (0.91-1.41)  Rectal cancer:  1.26 (0.87-1.83) | age, sex, total energy, education, BMI, smoking, physical activity, use of hormone therapy (women), multivitamin use, aspirin use, beer, wine, liquor, fruits, vegetable, high-fiber grain foods | 8 |
| Larsson 2005 Sweden[48] | co | Colorectal cancer:  733/61433  Colon cancer:  234/61433  Rectal cancer:  230/61433 | 1987-1990 | FFQ-67 | Red meat  Processed meat | Quartile | Colorectal cancer:  RD: 1.32 (1.03-1.68)  PD: 1.07 (0.85-1.33)  Colon cancer:  RD: 1.03 (0.67-1.60)  PD: 1.39 90.86-2.24）  Rectal cancer:  RD: 1.28 (0.83-1.98)  PD: 0.90 (0.60-1.34) | age, BMI, educational and intakes of total energy, alcohol, saturated fat, calcium, folate, fruits, vegetables, whole-grain foods | 9 |
| Norat 2005 Europe[49] | co | Colorectal cancer:  1329/478040  Colon cancer:  855/478040  Rectal cancer:  474/478040 | 1992-1998 | FFQ-266 | Red meat  Processed meat | Quintile | Colorectal cancer:  RD: 1.17 (0.92-1.49)  PD: 1.42 (1.09-1.86)  Colon cancer:  RD: 1.20 (0.88-1.61)  PD: 1.30 (0.91-1.85)  Rectal cancer:  RD: 1.13 (0.74-1.71)  PD: 1.62 (1.04-2.51) | sex, energy from fat, energy from -nonfat sources except alcohol, height, weight, current alcohol intake, physical activity, smoking, fiber intake | 9 |
| Oba 2006 Japan[50] | co | Colon cancer:  213/30221 | 1992-2000 | FFQ-169 | Red meat  Processed meat | Tertile | Colon cancer:  1.03 (0.64-1.66)  1.98 (1.24-3.16) | age, height, BMI, smoking, alcohol intake, and physical activity | 7 |
| Sato 2006 Japan[51] | co | Colorectal cancer:  396/41835  Colon cancer:  240/41835  Rectal cancer:  159/41835 | 1990  Jun-Aug | FFQ-40 | Pork (excluding ham or sausage)  Ham/sausage | Quartile | Colorectal cancer:  RD: 1.13 (0.79-1.62)  PD: 1.02 (0.77-1.36)  Colon cancer:  RD: 1.46 (0.81-2.02)  PD: 0.75 (0.45-1.25)  Rectal cancer:  RD: 0.74 (0.39-1.42)  PD: 1.10 (0.60-2.02) | sex, age, smoking, alcohol, BMI, education, family history, walking, consumption of fat, calcium, and dietary fibre | 8 |
| Cross 2007 USA[52] | co | Colorectal cancer:  5107/494036  Colon cancer:  1995/494036  Rectal cancer:  724/494036 | 1995-1996 | FFQ-124 | Processed meat | Quintile | Colorectal cancer  1.20 (1.09-1.32)  Colon cancer  1.18 (1.06-1.31)  Rectal cancer:  1.24 (1.03-1.49) | age, sex, BMI, education, ethnicity, smoking, alcohol, physical activity, fruit and vegetables, saturated fat, and calories intake | 9 |
| Butler 2008 Singapore[53] | co | 961/61321 | 1998-2005 | FFQ-165 | Red meat | Quartile | 1.01 (0.82-1.26) | age, interview year, smoking, BMI, alcohol, education, physical activity, first-degree relative diagnosed with CRC, total daily energy intake | 8 |
| Lee 2009 China[54] | co | Colorectal cancer:  394/73224  Colon cancer:  236/73224  Rectal cancer:  158/73224 | 1997-2000 | FFQ-NS | Red meat  Salted meat | Quintile | Colorectal cancer:  RD: 0.80 (0.60-1.10)  PD: 1.10 (0.80-1.40)  Colon cancer:  RD: 0.90 (0.50-1.50)  PD: 1.10 (0.80-1.51)  Rectal cancer:  RD: 0.60 (0.30-1.10)  PD: 0.90 (0.60-1.35) | age, education, income, survey season, tea, NSAID use, energy intake, and fiber intake | 8 |
| Cross 2010 USA[55] | co | 2719/300948 | 1996-2003 | FFQ-124 | Unprocessed red meat | Quintile | 1.13 (0.98-1.30) | gender, education, BMI, smoking, total energy intake, fiber and dietary calcium, white meat intake | 8 |
| Takachi 2011 Japan[56] | co | Colon cancer:  788/80658  Rectal cancer:  357/80658 | 1998-2006 | FFQ-138 | Processed meat | Quintile | Colon cancer:  1.27 (0.85-1.90)  Rectal cancer:  0.70 (0.45-1.09) | age, Public Health Center area, BMI, smoking, alcohol, physical activity, diabetes, energy intake, calcium, vitamin D/B6, folate, dietary fiber, dried and salted fish | 8 |
| Ollberding 2012 USA[57] | co | 3404/131763 | 1993-1996  1999-2000 | FFQ-NS | Red meat  Processed meat | Quintile | 0.98 (0.87-1.10)  1.06 (0.94-1.19) | family history of colorectal polyp, BMI, smoking, NSAID, alcohol, physical activity, history of diabetes, hormone replacement therapy use, total calories, dietary fiber, calcium, folate, vitamin D | 8 |
| Bernstein 2015 USA[58] | co | Colorectal cancer:  2731/134497  Colon cancer:  2962/134497  Rectal cancer:  2207/134497 | 1990-2010 | FFQ-131 | Processed meat | Quintile | Colorectal cancer:  1.15 (1.01-1.32)  Colon cancer:  1.36 (1.09-1.69)  Rectal cancer:  1.18 (0.89-1.57) | age, family history, prior lower gastrointestinal endoscopy, smoking, BMI, physical activity, total caloric intake, alcohol, and energy-adjusted intake of folate, calcium, vitamin D and total fiber | 7 |
| Gilsing 2015 Netherlands[59] | co | Colorectal cancer:  437/10210  Colon cancer:  307/10210  Rectal cancer:  102/10210 | 1986-2006 | FFQ-150 | Fresh red meat  Processed meat | Quartile | Colorectal cancer:  RD: 1.20 (0.83-1.74)  PD: 1.24 (0.91-1.69)  Colon cancer:  RD: 1.08 (0.70-1.64)  PD: 1.17 (0.81-1.69)  Rectal cancer:  RD: 1.56 (0.58-4.23)  PD: 1.88 (0.94-3.75) | age, sex, total energy, smoking, alcohol, BMI, non-occupational physical activity, education | 8 |
| Le 2016 USA[60] | co | Colorectal cancer:  1208/95490  Colon cancer:  555/95490  Rectal cancer:  246/95490 | 1996-2010 | FFQ and CQ | Red meat | Quintile | Colorectal cancer:  1.20 (0.99-1.45)  Colon cancer:  1.11 (1.02-1.21)  Rectal cancer:  1.10 (0.95-1.27) | age, family history of CRC in first degree relatives, prior lower gastrointestinal endoscopy, smoking, physical activity, aspirin or NSAID use, total caloric intake, alcohol | 9 |

RD: red meat; PD: processed meat; CRC: colorectal cancer; cc: case-control; co: cohort; RRs/ORs: relative risks/odds ratios; 95% CI: 95% confidence intervals; FFQ: food frequency questionnaire; BMI: body mass index; NSAID: nonsteroidal anti-inflammatory drug; SES: socio-economic status; IBD: inflammatory bowel disease; PHQ: personal history questionnaire; DHQ: diet history questionnaire. RFQ: risk factor questionnaire. CQ: cooking questionnaire.

**REFERENCES**

1. Lee HP, Gourley L, Duffy SW, Esteve J, Lee J, Day NE. Colorectal cancer and diet in an Asian population--a case-control study among Singapore Chinese. Int J Cancer. 1989; 43:1007-1016.

2. Steinmetz KA, Potter JD. Food-group consumption and colon cancer in the Adelaide Case-Control Study. II. Meat, poultry, seafood, dairy foods and eggs. Int J Cancer. 1993; 53:720-727.

3. Muscat JE, Wynder EL. The consumption of well-done red meat and the risk of colorectal cancer. Am J Public Health. 1994; 84:856-858.

4. Levi F, Pasche C, La Vecchia C, Lucchini F, Franceschi S. Food groups and colorectal cancer risk. Br J Cancer. 1999; 79:1283-1287.

5. Kampman E, Slattery ML, Bigler J, Leppert M, Samowitz W, Caan BJ, Potter JD. Meat consumption, genetic susceptibility, and colon cancer risk: a United States multicenter case-control study. Cancer Epidemiol Biomarkers Prev. 1999; 8:15-24.

6. Evans RC, Fear S, Ashby D, Hackett A, Williams E, Van Der Vliet M, Dunstan FD, Rhodes JM. Diet and colorectal cancer: an investigation of the lectin/galactose hypothesis. Gastroenterology. 2002; 122:1784-1792.

7. Le Marchand L, Hankin JH, Pierce LM, Sinha R, Nerurkar PV, Franke AA, Wilkens LR, Kolonel LN, Donlon T, Seifried A, Custer LJ, Lum-Jones A, Chang W. Well-done red meat, metabolic phenotypes and colorectal cancer in Hawaii. Mutat Res. 2002; 506-507:205-214.

8. Seow A, Quah SR, Nyam D, Straughan PT, Chua T, Aw TC. Food groups and the risk of colorectal carcinoma in an Asian population. Cancer. 2002; 95:2390-2396.

9. Tiemersma EW, Kampman E, Bueno DM, Bunschoten A, van Schothorst EM, Kok FJ, Kromhout D. Meat consumption, cigarette smoking, and genetic susceptibility in the etiology of colorectal cancer: results from a Dutch prospective study. Cancer Causes Control. 2002; 13:383-393.

10. Chiu BC, Ji BT, Dai Q, Gridley G, McLaughlin JK, Gao YT, Fraumeni JJ, Chow WH. Dietary factors and risk of colon cancer in Shanghai, China. Cancer Epidemiol Biomarkers Prev. 2003; 12:201-208.

11. Levi F, Pasche C, Lucchini F, Bosetti C, La Vecchia C. Processed meat and the risk of selected digestive tract and laryngeal neoplasms in Switzerland. Ann Oncol. 2004; 15:346-349.

12. Murtaugh MA, Ma KN, Sweeney C, Caan BJ, Slattery ML. Meat consumption patterns and preparation, genetic variants of metabolic enzymes, and their association with rectal cancer in men and women. J Nutr. 2004; 134:776-784.

13. Navarro A, Munoz SE, Lantieri MJ, Del PD, Cristaldo PE, de Fabro SP, Eynard AR. Meat cooking habits and risk of colorectal cancer in Cordoba, Argentina. Nutrition. 2004; 20:873-877.

14. Turner F, Smith G, Sachse C, Lightfoot T, Garner RC, Wolf CR, Forman D, Bishop DT, Barrett JH. Vegetable, fruit and meat consumption and potential risk modifying genes in relation to colorectal cancer. Int J Cancer. 2004; 112:259-264.

15. Chan AT, Tranah GJ, Giovannucci EL, Willett WC, Hunter DJ, Fuchs CS. Prospective study of N-acetyltransferase-2 genotypes, meat intake, smoking and risk of colorectal cancer. Int J Cancer. 2005; 115:648-652.

16. Murtaugh MA, Sweeney C, Ma KN, Caan BJ, Slattery ML. The CYP1A1 genotype may alter the association of meat consumption patterns and preparation with the risk of colorectal cancer in men and women. J Nutr. 2005; 135:179-186.

17. Kuriki K, Hirose K, Matsuo K, Wakai K, Ito H, Kanemitsu Y, Hirai T, Kato T, Hamajima N, Takezaki T, Suzuki T, Saito T, Tanaka R, et al. Meat, milk, saturated fatty acids, the Pro12Ala and C161T polymorphisms of the PPARgamma gene and colorectal cancer risk in Japanese. Cancer Sci. 2006; 97:1226-1235.

18. Kimura Y, Kono S, Toyomura K, Nagano J, Mizoue T, Moore MA, Mibu R, Tanaka M, Kakeji Y, Maehara Y, Okamura T, Ikejiri K, Futami K, et al. Meat, fish and fat intake in relation to subsite-specific risk of colorectal cancer: the Fukuoka Colorectal Cancer Study. Cancer Sci. 2007; 98:590-597.

19. Girard H, Butler LM, Villeneuve L, Millikan RC, Sinha R, Sandler RS, Guillemette C. UGT1A1 and UGT1A9 functional variants, meat intake, and colon cancer, among Caucasians and African-Americans. Mutat Res. 2008; 644:56-63.

20. Hu J, La Vecchia C, DesMeules M, Negri E, Mery L. Meat and fish consumption and cancer in Canada. Nutr Cancer. 2008; 60:313-324.

21. Saebo M, Skjelbred CF, Brekke LK, Bowitz LI, Hagen PC, Johnsen E, Tveit KM, Kure EH. CYP1A2 164 A-->C polymorphism, cigarette smoking, consumption of well-done red meat and risk of developing colorectal adenomas and carcinomas. Anticancer Res. 2008; 28:2289-2295.

22. Aune D, De Stefani E, Ronco A, Boffetta P, Deneo-Pellegrini H, Acosta G, Mendilaharsu M. Meat consumption and cancer risk: a case-control study in Uruguay. Asian Pac J Cancer Prev. 2009; 10:429-436.

23. Nayak SP, Sasi MP, Sreejayan MP, Mandal S. A case-control study of roles of diet in colorectal carcinoma in a South Indian population. Asian Pac J Cancer Prev. 2009; 10:565-568.

24. Williams CD, Satia JA, Adair LS, Stevens J, Galanko J, Keku TO, Sandler RS. Dietary patterns, food groups, and rectal cancer risk in Whites and African-Americans. Cancer Epidemiol Biomarkers Prev. 2009; 18:1552-1561.

25. Squires J, Roebothan B, Buehler S, Sun Z, Cotterchio M, Younghusband B, Dicks E, Mclaughlin JR, Parfrey PS, Wang PP. Pickled meat consumption and colorectal cancer (CRC): a case-control study in Newfoundland and Labrador, Canada. Cancer Causes Control. 2010; 21:1513-1521.

26. Spencer EA, Key TJ, Appleby PN, Dahm CC, Keogh RH, Fentiman IS, Akbaraly T, Brunner EJ, Burley V, Cade JE, Greenwood DC, Stephen AM, Mishra G, et al. Meat, poultry and fish and risk of colorectal cancer: pooled analysis of data from the UK dietary cohort consortium. Cancer Causes Control. 2010; 21:1417-1425.

27. Wang H, Yamamoto JF, Caberto C, Saltzman B, Decker R, Vogt TM, Yokochi L, Chanock S, Wilkens LR, Le Marchand L. Genetic variation in the bioactivation pathway for polycyclic hydrocarbons and heterocyclic amines in relation to risk of colorectal neoplasia. Carcinogenesis. 2011; 32:203-209.

28. Williams CD, Satia JA, Adair LS, Stevens J, Galanko J, Keku TO, Sandler RS. Associations of red meat, fat, and protein intake with distal colorectal cancer risk. Nutr Cancer. 2010; 62:701-709.

29. Hu J, La Vecchia C, Morrison H, Negri E, Mery L. Salt, processed meat and the risk of cancer. Eur J Cancer Prev. 2011; 20:132-139.

30. De Stefani E, Boffetta P, Ronco AL, Deneo-Pellegrini H, Correa P, Acosta G, Mendilaharsu M, Luaces ME, Silva C. Processed meat consumption and risk of cancer: a multisite case-control study in Uruguay. Br J Cancer. 2012; 107:1584-1588.

31. Di Maso M, Talamini R, Bosetti C, Montella M, Zucchetto A, Libra M, Negri E, Levi F, La Vecchia C, Franceschi S, Serraino D, Polesel J. Red meat and cancer risk in a network of case-control studies focusing on cooking practices. Ann Oncol. 2013; 24:3107-3112.

32. Miller PE, Lazarus P, Lesko SM, Cross AJ, Sinha R, Laio J, Zhu J, Harper G, Muscat JE, Hartman TJ. Meat-related compounds and colorectal cancer risk by anatomical subsite. Nutr Cancer. 2013; 65:202-226.

33. Steck SE, Butler LM, Keku T, Antwi S, Galanko J, Sandler RS, Hu JJ. Nucleotide excision repair gene polymorphisms, meat intake and colon cancer risk. Mutat Res. 2014; 762:24-31.

34. Abu MS, Tayyem RF, Shehadah I, Bawadi HA, Agraib LM, Bani-Hani KE, Al-Jaberi T, Al-Nusairr M. Food groups and the risk of colorectal cancer: results from a Jordanian case-control study. Eur J Cancer Prev. 2015; 24:313-320.

35. Chun YJ, Sohn SK, Song HK, Lee SM, Youn YH, Lee S, Park H. Associations of colorectal cancer incidence with nutrient and food group intakes in Korean adults: a case-control study. Clin Nutr Res. 2015; 4:110-123.

36. Joshi AD, Kim A, Lewinger JP, Ulrich CM, Potter JD, Cotterchio M, Le Marchand L, Stern MC. Meat intake, cooking methods, dietary carcinogens, and colorectal cancer risk: findings from the Colorectal Cancer Family Registry. Cancer Med. 2015; 4:936-952.

37. Espejo-Herrera N, Gracia-Lavedan E, Boldo E, Aragones N, Perez-Gomez B, Pollan M, Molina AJ, Fernandez T, Martin V, La Vecchia C, Bosetti C, Tavani A, Polesel J, et al. Colorectal cancer risk and nitrate exposure through drinking water and diet. Int J Cancer. 2016; 139:334-346.

38. Bostick RM, Potter JD, Kushi LH, Sellers TA, Steinmetz KA, McKenzie DR, Gapstur SM, Folsom AR. Sugar, meat, and fat intake, and non-dietary risk factors for colon cancer incidence in Iowa women (United States). Cancer Causes Control. 1994; 5:38-52.

39. Kato I, Akhmedkhanov A, Koenig K, Toniolo PG, Shore RE, Riboli E. Prospective study of diet and female colorectal cancer: the New York University Women's Health Study. Nutr Cancer. 1997; 28:276-281.

40. Singh PN, Fraser GE. Dietary risk factors for colon cancer in a low-risk population. Am J Epidemiol. 1998; 148:761-774.

41. Knekt P, Jarvinen R, Dich J, Hakulinen T. Risk of colorectal and other gastro-intestinal cancers after exposure to nitrate, nitrite and N-nitroso compounds: a follow-up study. Int J Cancer. 1999; 80:852-856.

42. Pietinen P, Malila N, Virtanen M, Hartman TJ, Tangrea JA, Albanes D, Virtamo J. Diet and risk of colorectal cancer in a cohort of Finnish men. Cancer Causes Control. 1999; 10:387-396.

43. Jarvinen R, Knekt P, Hakulinen T, Rissanen H, Heliovaara M. Dietary fat, cholesterol and colorectal cancer in a prospective study. Br J Cancer. 2001; 85:357-361.

44. Flood A, Velie EM, Sinha R, Chaterjee N, Lacey JJ, Schairer C, Schatzkin A. Meat, fat, and their subtypes as risk factors for colorectal cancer in a prospective cohort of women. Am J Epidemiol. 2003; 158:59-68.

45. Lin J, Zhang SM, Cook NR, Lee IM, Buring JE. Dietary fat and fatty acids and risk of colorectal cancer in women. Am J Epidemiol. 2004; 160:1011-1022.

46. English DR, MacInnis RJ, Hodge AM, Hopper JL, Haydon AM, Giles GG. Red meat, chicken, and fish consumption and risk of colorectal cancer. Cancer Epidemiol Biomarkers Prev. 2004; 13:1509-1514.

47. Chao A, Thun MJ, Connell CJ, McCullough ML, Jacobs EJ, Flanders WD, Rodriguez C, Sinha R, Calle EE. Meat consumption and risk of colorectal cancer. JAMA. 2005; 293:172-182.

48. Larsson SC, Rafter J, Holmberg L, Bergkvist L, Wolk A. Red meat consumption and risk of cancers of the proximal colon, distal colon and rectum: the Swedish Mammography Cohort. Int J Cancer. 2005; 113:829-834.

49. Norat T, Bingham S, Ferrari P, Slimani N, Jenab M, Mazuir M, Overvad K, Olsen A, Tjonneland A, Clavel F, Boutron-Ruault MC, Kesse E, Boeing H, et al. Meat, fish, and colorectal cancer risk: the European Prospective Investigation into cancer and nutrition. J Natl Cancer Inst. 2005; 97:906-916.

50. Oba S, Shimizu N, Nagata C, Shimizu H, Kametani M, Takeyama N, Ohnuma T, Matsushita S. The relationship between the consumption of meat, fat, and coffee and the risk of colon cancer: a prospective study in Japan. Cancer Lett. 2006; 244:260-267.

51. Sato Y, Nakaya N, Kuriyama S, Nishino Y, Tsubono Y, Tsuji I. Meat consumption and risk of colorectal cancer in Japan: the Miyagi Cohort Study. Eur J Cancer Prev. 2006; 15:211-218.

52. Cross AJ, Leitzmann MF, Gail MH, Hollenbeck AR, Schatzkin A, Sinha R. A prospective study of red and processed meat intake in relation to cancer risk. PLoS Med. 2007; 4:e325.

53. Butler LM, Wang R, Koh WP, Yu MC. Prospective study of dietary patterns and colorectal cancer among Singapore Chinese. Br J Cancer. 2008; 99:1511-1516.

54. Lee SA, Shu XO, Yang G, Li H, Gao YT, Zheng W. Animal origin foods and colorectal cancer risk: a report from the Shanghai Women's Health Study. Nutr Cancer. 2009; 61:194-205.

55. Cross AJ, Ferrucci LM, Risch A, Graubard BI, Ward MH, Park Y, Hollenbeck AR, Schatzkin A, Sinha R. A large prospective study of meat consumption and colorectal cancer risk: an investigation of potential mechanisms underlying this association. Cancer Res. 2010; 70:2406-2414.

56. Takachi R, Tsubono Y, Baba K, Inoue M, Sasazuki S, Iwasaki M, Tsugane S; Japan Public Health Center-Based Prospective Study Group. Red meat intake may increase the risk of colon cancer in Japanese, a population with relatively low red meat consumption. Asia Pac J Clin Nutr. 2011; 20:603-612.

57. Ollberding NJ, Wilkens LR, Henderson BE, Kolonel LN, Le Marchand L. Meat consumption, heterocyclic amines and colorectal cancer risk: the Multiethnic Cohort Study. Int J Cancer. 2012; 131:E1125-E1133.

58. Bernstein AM, Song M, Zhang X, Pan A, Wang M, Fuchs CS, Le N, Chan AT, Willett WC, Ogino S, Giovannucci EL, Wu K. Processed and unprocessed red meat and risk of colorectal cancer: analysis by tumor location and modification by time. PLoS One. 2015; 10:e135959.

59. Gilsing AM, Schouten LJ, Goldbohm RA, Dagnelie PC, van den Brandt PA, Weijenberg MP. Vegetarianism, low meat consumption and the risk of colorectal cancer in a population based cohort study. Sci Rep. 2015; 5:13484.

60. Le NT, Michels FA, Song M, Zhang X, Bernstein AM, Giovannucci EL, Fuchs CS, Ogino S, Chan AT, Sinha R, Willett WC, Wu K. A prospective analysis of meat mutagens and colorectal cancer in the nurses' health study and health professionals follow-up study. Environ Health Perspect. 2016; 124:1529-1536.
